# Composites and Materials Prepared from Boron Cluster Anions and Carboranes

**DOI:** 10.3390/ma16186099

**Published:** 2023-09-06

**Authors:** Varvara V. Avdeeva, Svetlana E. Nikiforova, Elena A. Malinina, Igor B. Sivaev, Nikolay T. Kuznetsov

**Affiliations:** 1 Kurnakov Institute of General and Inorganic Chemistry, Russian Academy of Sciences, 31 Leninskii Av., Moscow 119991, Russia; korolencko0110@yandex.ru (S.E.N.); malinina@igic.ras.ru (E.A.M.); ntkuz@igic.ras.ru (N.T.K.); 2 Nesmeyanov Institute of Organoelement Compounds, Russian Academy of Sciences, 28 Vavilov Str., Moscow 119991, Russia; sivaev@ineos.ac.ru; 3 Basic Department of Chemistry of Innovative Materials and Technologies, Plekhanov Russian University of Economics, 36 Stremyannyi Line, Moscow 117997, Russia

**Keywords:** boron clusters, carborane, composites, borides

## Abstract

Here, we present composites and materials that can be prepared starting with boron hydride cluster compounds (decaborane, decahydro-*closo*-decaborate and dodecahydro-*closo*-dodecaborate anions and carboranes). Recent examples of their utilization as boron protective coatings including using them to synthesize boron carbide, boron nitride, metal borides, metal-containing composites, and neutron shielding materials are discussed. The data are generalized demonstrate the versatile application of materials based on boron cluster anions and carboranes in various fields.

## 1. Introduction

The driving force behind the intensive development of the chemistry of boron hydride cluster compounds in the 1950s was their use as high-energy materials [1,2,3]. This topic is still discussed, although it attracts less attention [4,5,6,7,8,9,10,11,12,13,14]. Currently, the most attention is paid to the use of boron cluster compounds in medicine [15,16,17,18,19,20,21,22,23,24,25,26,27]. As for the use of boron clusters in materials science, complex molecular and supramolecular structures, such as nanocars and nanotrains [28,29,30,31], molecular machines, and switches [32,33,34,35,36], MOFs [37,38,39,40,41,42,43,44,45,46], etc., attract the most attention. However, there is another less spectacular but no less important direction of research on the use of boron cluster compounds in the science of materials. This direction of research consists of the thermal decomposition of various boron clusters with the formation of boron-containing coatings and ceramic materials, which is somewhat reminiscent of their use as high-energy materials, since in this case a complex molecular structure is also converted into simple molecules.

**Figure 1:**
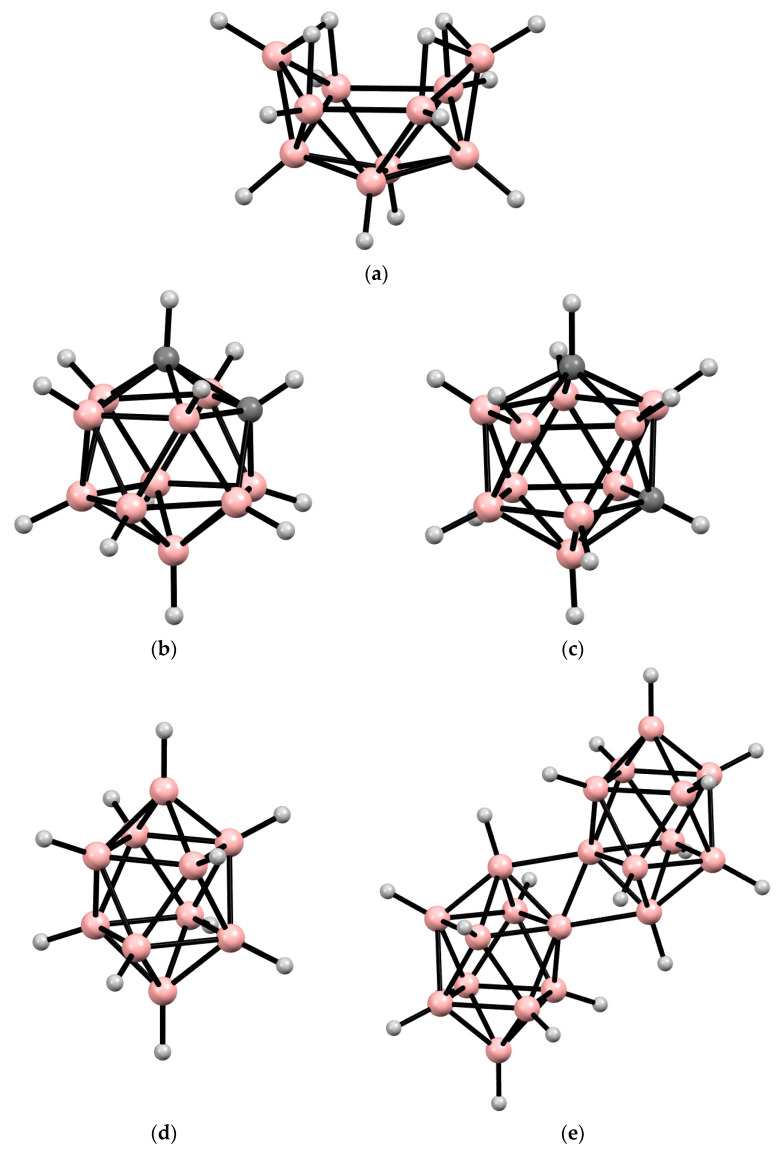
Idealized structures of decaborane(14) B_10_H_14_ (**a**), *ortho*-carborane 1,2-C_2_B_10_H_12_ (**b**), *meta*-carborane 1,7-C_2_B_10_H_12_ (**c**), decahydro-*closo*-decaborate anion [B_10_H_10_]^2−^ (**d**), and octadecahydro-*conjucto*-eicosoborate anion [*trans*-B_20_H_18_]^2−^ (**e**).

In this contribution to the field, we present an attempt to consider the use of boron clusters to obtain various boron-containing materials, including boron-containing coatings and films, nanostructured boron carbide and metal borides, and others. The most readily available decaborane(14) B_10_H_14_, *ortho*- and *meta*-carboranes 1,2-C_2_B_10_H_12_ and 1,2-C_2_B_10_H_12_, decahydro-*closo*-decaborate [B_10_H_10_]^2−^ and octadecahydro-*conjucto*-eicosaborate [*trans*-B_20_H_18_]^2−^ anions (Figure 1) are considered as the boron clusters. Decaborane(14) can be readily prepared in a two-step procedure from sodium tetrahydroborate NaBH_4_ [47]. The well-known *ortho*-carborane is obtained by introducing the acetylene molecule into the open boron cage of decaborane(14), and its thermal isomerization leads to *meta*-carborane [48]. The decahydro-*closo*-decaborate anion [49,50,51] is formed by heating decaborane(14) in the presence of triethylamine, and its mild oxidation results in the octadecahydro-*conjucto*-eicosaborate anion [52,53].

## 2. Decaborane as Boron Source for Boron-Containing Materials

Metal Organic Chemical Vapor Deposition (MOCVD) is widely used for creating high-purity crystalline semiconducting thin films and micro/nano structures for microelectronics [54,55,56,57]. Therefore, it is not surprising that volatile boron hydrides such as decaborane have been proposed for the preparation of various boron-containing coatings. At first glance, it may seem that for these purposes it is more convenient to use other more available and cheap volatile boron compounds, such as BCl_3_, BF_3_ or diborane. However, one drawback of MOCVD is the aggressive, toxic and explosive nature of the precursor gases, which makes them difficult to use in small research laboratories. This fully applies to both aggressive and corrosive boron halides and highly toxic and flammable diborane. Therefore, decaborane B_10_H_14_, despite its higher cost and toxicity [58,59], is in many respects a more convenient source of boron for these purposes. Furthermore, decaborane has advantages as a source material for boron coating because high-purity decaborane is easy to obtain with a sublimation purification process.

Amorphous boron films of 0.1–1.5 μm thickness have been prepared on sapphire, silicon, and tantalum as substrates by the pyrolysis of decaborane in the molecular flow region (≤10^−4^ torr) and in a temperature range of 350–1200 °C. It is found that the deposition rate of the boron films is proportional to the decaborane partial pressure and the substrate temperature. The electrical conductivities vary from 3 × 10^−5^ S cm^−1^ at 77 K to 30 S cm^−1^ at 1000 K, and the activation energy is 1.07 eV in the intrinsic temperature range (700–1000 K). The maximum value of thermoelectric power is about 420 μV deg^−1^ at 700 K, and its polarity is positive between 500 and 1000 K [60]. The preparation of polycrystalline α-rhombohedral boron films by pyrolysis of decaborane has also been reported [61].

Boron coating also can be obtained by the plasma-assisted enhanced chemical vapor deposition (PECVD) of decaborane [62,63]. In particular, decaborane has been proposed as a boron source for the boronization of JT-60U Tokamak to reduce the influx of impurities during plasma discharge [64]. The deposition of boron films on polished *p*-type Si(111) surface by synchrotron-radiation-induced chemical vapor deposition (SR-CVD) of decaborane was reported [65].

When using ammonia or dinitrogen as additives, the chemical vapor deposition of decaborane can be used to obtain thin films of boron nitride [66], nanosheets [67], and nanotubes [68], competing with borazine. In particular, boron nitride nanotubes (BNNTs) grown at 1200–1300 °C from decaborane were double- and multiwalled, with the double-walled nanotubes having ~2 nm inner diameters and the multiwalled nanotubes (~10 walls) having ~4–5 nm inner diameters and ~12–14 nm outer diameters. The nanotubes grown at 1300 °C were longer, averaging ~0.6 μm, whereas those grown at 1200 °C had average lengths of ~0.2 μm [68].

The pyrolysis of decaborane can also be used to prepare boron nanoparticles [69] and microcrystals [70,71]. α-Tetragonal boron crystals were obtained at a pressure of 8–9 GPa and temperatures in the range 1100–1600 °C, while β-rhombohedral boron crystals grow at 3 GPa and 1200 °C [70]. The α-tetragonal boron crystals synthesized demonstrate semiconducting properties of conductivity with the energy gap *E*_g_ ≈ 1.5 eV [71].

The chemical vapor deposition of decaborane was also used to prepare various metal boride thin films, including nickel [72], strontium [73], gadolinium [74], neodymium [75], and ytterbium [76,77].

A synthetic route to metal borides TiB_2_, ZrBz_2_, HfB_2_, NbB_2_, and TaB_2_ by heating the decaborane-pimelonitrilium polymer [-6-B_10_H_12_-(NC(CH_2_)_5_CN)]*_n_*- and the corresponding finely dispersed metal oxides above 1400 °C was proposed. The metal boride powders were found to be highly crystalline, with grain sizes dependent on processing temperatures [78].

Various boron-carbide-containing materials were prepared using various decaborane-based single-molecular precursors, such as [μ-6,6′-(CH_2_)_6_-(B_10_H_13_)_2_] [79,80], [μ-6,6′-(1′,5′-cyclooctyl)-(B_10_H_13_)_2_] [81], [μ-6,6′-(2′,5′-norbornenyl)-(B_10_H_13_)_2_] [81], or polymers, including [-6-B_10_H_12_-Ph_2_POPPh_2_]*_n_*- [82], [-6-B_10_H_12_-(CH_2_)_6_]*_n_*- [83,84], [-6-B_10_H_12_-(2′,5′-norbornenyl)]*_n_*- [81,83,84,85,86,87,88,89], [-6-B_10_H_12_-(1′,5′-cyclooctenyl)]*_n_*- [81,83], and [-6-B_10_H_12_-(1′,4′-cyclooctenyl)]*_n_*- [81,83].

**Figure 2:**
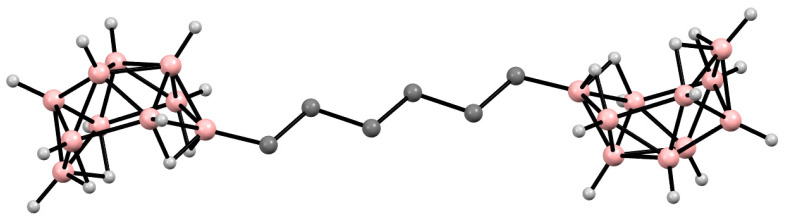
Solid-state structure of [μ-6,6′-(CH_2_)_6_-(B_10_H_13_)_2_]. Hydrogen atoms of organic substituents are omitted for clarity.

**Figure 3:**
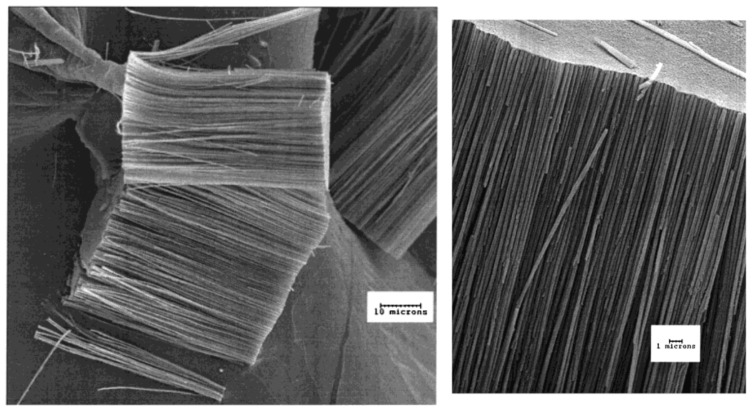
SEM images of aligned boron carbide nanofibers obtained upon pyrolysis of [μ-6,6′-(CH_2_)_6_-(B_10_H_13_)_2_] at 1025 °C. Reprinted with permission from Ref. [79]. Copyright (2000) the American Chemical Society.

In particular, [μ-6,6′-(CH_2_)_6_-(B_10_H_13_)_2_] (Figure 2) appears to be an ideal precursor for the synthesis of boron carbide nanofibers (Figure 3) using the templating technique: (i) it is readily synthesized in large amounts using the Ti-catalyzed reaction [90]; (ii) it contains no other ceramic-forming elements and has a desirable boron-to-carbon ratio, thus yielding boron-rich boron carbide compositions upon pyrolysis; (iii) it is stable as a liquid, allowing it to be absorbed into the membrane without decomposition; and (iv) upon pyrolysis, it undergoes a cross-linking reaction at relatively low temperatures (220 °C), which slows the loss of material by volatilization, thereby generating high ceramic and chemical yields [79].

The bis(decaboranyl)-hexane precursor [μ-6,6′-(CH_2_)_6_-(B_10_H_13_)_2_] can also be used for the preparation of ordered mesoporous boron carbide materials with high specific surface areas up to 778 m^2^/g and hexagonal pore arrangement symmetries [80].

It should be noted that an alternative possibility of using pentaborane B_5_H_9_ instead of decaborane to obtain boron carbide compositions was previously considered [91,92,93]; however, after the destruction of pentaborane stocks stored since the 1960s [94], this aim was abandoned.

**Figure 4:**
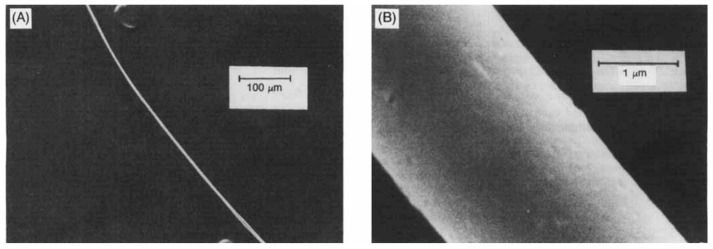
SEM images of ceramic fibers at different scale (**A**,**B**) derived from [-6-B_10_H_12_-(ethylenediamine)]*_n_*- by pyrolysis at 1000 °C under argon. Reprinted with permission from Ref. [87]. Copyright (1988) the American Ceramic Society.

The B_4_C/BN-containing ceramic materials can be prepared using the pyrolysis of polymeric Lewis base adducts of decaborane [-6-B_10_H_12_-(diamine)]*_n_*- (diamine is ethylenediamine, 1,1-dimethylethylenediamine, 1,1,2,2-tetramethylethylenediamine) [95,96]. In particular, the [-6-B_10_H_12_-(ethylenediamine)]*_n_*-polymer fibers upon pyrolysis at 1000 °C in an argon atmosphere retain their shape and give black ceramic fibers with a diameter of 3 to 5 μm, which have a round shape, a smooth surface, and no obvious major flaws (Figure 4). Other [-6-B_10_H_12_-(diamine)]*_n_*- polymers were capable of forming fibers. The polymers derived from 1,1,2,2-tetramethylethylenediamine and from the 85/15 1,1-dimethylethylenediamine/1,1,2,2-tetramethylethylenediamine mixture melt when heated (mp 246–250 °C and 222–225 °C, respectively) and may be suitable for melt-spinning [87].

Low-crystalline boron nitride was prepared by the reaction of triammoniadecaborane B_10_H_14_·3NH_3_ and hydrazine or ammonia at 125 MPa and 650–700 °C. The prepared low-crystalline boron nitride passed into cubic boron nitride at 1200–1300 °C and 6.5 GPa in the presence of 20 mol.% AINas a catalyst [97,98].

## 3. Carborane as a Boron Source for Boron-Containing Materials

Chemical vapor deposition methods are widely used for the manufacture of boron carbide films due to the better controlled deposition process and the high-quality boron carbide production. Mixtures of boron trichloride, methane, and hydrogen are usually used for the CVD of boron carbide films. Since chlorides are highly dangerous and the synthesis process requires high temperatures, the replacement of BCl_3_ with organoboranes has become a trend in recent years. At first it seemed that small organoboron molecules such as trimethylboron and triethylboron could be a good alternative, but they proved to be overly reactive. Taking into account that *ortho*-carborane [H_2_C_2_B_10_H_10_] provides a suitable ratio of B and C from a single molecular source, it seems to be an attractive source for preparing boron carbide materials.

**Figure 5:**
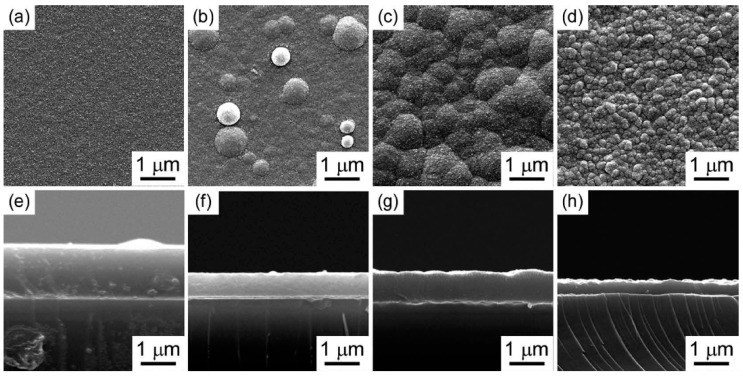
Surface and cross-section SEM images of boron carbide film prepared at T_dep_ = 900 °C, P_tot_ = 100 Pa (**a**,**e**), T_dep_ = 900 °C, P_tot_ = 50,000 Pa (**b**,**f**), T_dep_ = 1100 °C, P_tot_ = 5000 Pa (**c**,**g**), T_dep_ = 1200 °C, P_tot_ = 50,000 Pa (**d**,**h**). Reprinted from Ref. [112].

Semiconducting boron carbide represents a new class of materials with potential applications in neutron detection because ^10^B has a high cross-section (approximately 3800 barns) for neutrons at lower energies (~25 meV), based on the ^10^B(*n*,α)^7^Li neutron capture reaction [99,100,101,102,103,104,105]. This aroused great interest in the fabrication of boron carbide films using the PECVD of *ortho*-carborane, and the effect of the process parameters, such as temperature and total pressure, on the composition, microstructure, morphology, and properties of the boron carbide films obtained were studied [106,107,108,109,110,111,112,113,114]. In particular, the boron carbide film prepared at low temperatures and pressures (T_dep_ = 900 °C and P_tot_ = 100 Pa) showed a comparatively flat morphology, whereas the boron carbide films prepared at low temperature and high pressure (T_dep_ = 900 °C and P_tot_ = 50,000 Pa) appeared as round bulges. The boron carbide films prepared at a high temperature and relatively low pressure (T_dep_ = 1100 °C and P_tot_ = 5000 Pa) exhibited a cauliflower-like surface, while the films prepared at high temperature and high pressure (T_dep_ = 1200 °C and P_tot_ = 50,000 Pa) exhibited a uniform granular surface (Figure 5) [112].

Semiconducting boron carbide films can be also prepared through the PECVD of *meta*-carborane, which differs from *ortho*-carborane only in the arrangement of carbon atoms in the icosahedral cage [115,116,117]. It was found that *meta*-carborane and *ortho*-carborane form self-doped *n*-type and *p*-type boron-carbides, respectively [115,116].

It was shown that neutron detectors and neutron voltaic devices, based on semiconducting boron carbides, contrary to most other electrical devices, may improve with some radiation exposure and are robust against radiation-induced device degradation and failure [104]. The main causes for the poor neutron detection device performance are the insufficiently thick depletion region of the device, the need for a thicker device to come closer to neutron opacity, and the need for better charge collection while maintaining low reverse bias leakage currents [118]. It was found that the semiconducting boron carbide prepared by PECVD of composites of *ortho*- and *meta-*carboranes and aromatic or heteroaromatic compounds [119,120,121,122,123,124,125] demonstrate improvements in both charge collection and reverse bias leakage currents, which is attributed to an increase in the hole carrier lifetimes.

The introduction of metallocenes Cp_2_M (M = Ni, Co, Fe, Mn) together with *ortho*- or *meta*-carboranes during the PECVD process results in the corresponding transition metal doping of semiconducting boron carbide films [126,127,128,129,130,131].

Another important area of using *ortho*- and *meta*-carboranes to create protective boron carbide coatings is the boronization of tokamaks. The plasma-chemical deposition of a protective coating on the first wall of a fusion device using a chemically active gas (precursor) remains to date one of the primary ways to protect plasma against cooling impurities. This method has proved effective and does not require the use of additional and expensive equipment. The use of a low-toxic and nonexplosive carborane for boronization made this method of obtaining boron-carbon coatings to be a quick, widely available, and relatively cheap one. The coatings obtained were found to be highly resistant to chemical erosion—the erosion coefficients were (5–6) × 10^−4^ at/ion regardless of temperature. The electrical resistance of the coating was high, and, depending on the deposition conditions, varied in the range of 10^9^–10^11^ Ω cm. The resistance of the coatings to the plasma impact was estimated using similar probes, which were examined after a certain number of working pulses. On all tokamaks, the coatings remained for several hundred pulses. The degradation of the coating correlates with the degradation of the plasma parameters [132,133,134,135,136].

**Figure 6:**
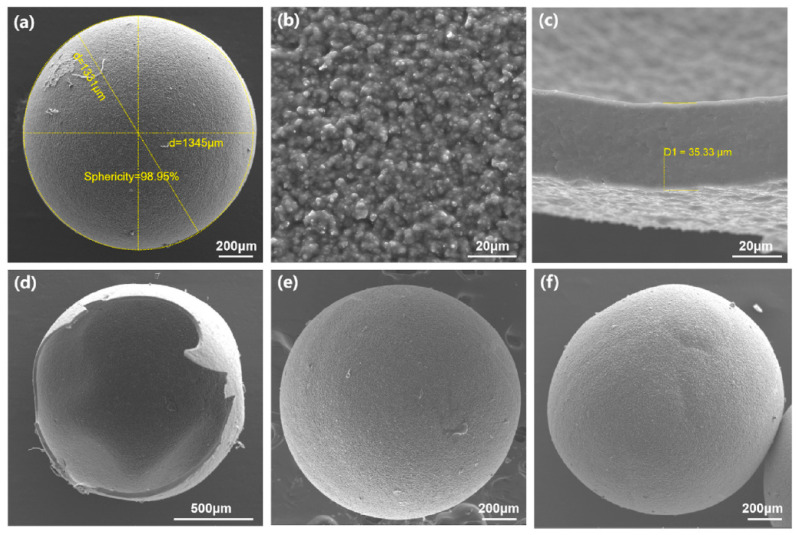
Hollow boron carbide microspheres prepared from poly(phenylene-*meta*-carborane)/polyacrylonitrile at different magnitude (**a**–**f**). Reprinted with permission from Ref. [141]. Copyright (2022) Elsevier.

The preparation of boron carbide through the pyrolysis of various carborane-containing polymers has been described [137,138,139,140]. The Ni-catalyzed polymerization of 1,2-bis(4-chloro-phenyl)-*ortho*-carborane leads to poly(phenylene-*ortho*-carborane). It was found that the heating of the polymer at 1000–1200 °C resulted in the crystallization of boron carbide, according to the X-ray powder diffraction studies [139]. The Ni-catalyzed polymerization of 1,7-bis(4-chlorophenyl)-*meta*-carborane produces poly(phenylene-*meta*-carborane), which can be used as a novel boron carbide precursor [140]. Due to its high ceramic yield, it can be used to prepare boron carbide ceramics with different shapes [141]. In particular, poly(phenylene-*meta*-carborane) was used to prepare the boron carbide hollow microsphere via slurry-coating and a method derived from a previous study. The poly(phenylene-*meta*-carborane)/polyacrylonitrile slurry was prepared and coated on a polyoxymethylene ball substrate. After air cross-linking, the substrate decomposition and heat-treatment at 1100 °C in argon atmosphere, hollow boron carbide microspheres with diameter of approximate 1.34 mm, and average shell thickness of 30 μm were obtained (Figure 6) [141].

**Figure 7:**
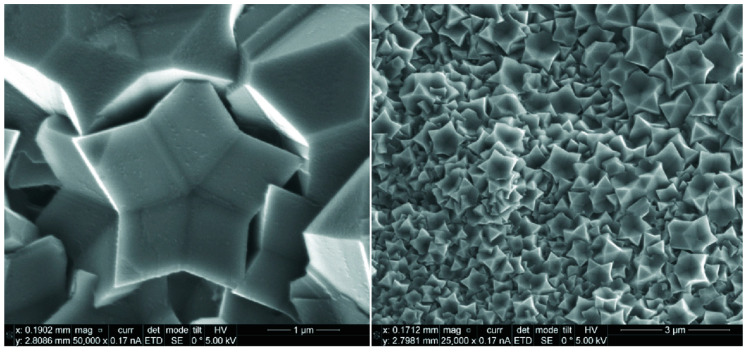
SEM images of star-shaped pentagonal boron carbide microcrystals prepared by thermobaric treatment of 1,7-bis(hydroxymethyl)-*meta*-carborane at 7 GPa and 1370 K. Reprinted from Ref. [142].

The star-shaped pentagonal microcrystals of boron carbide with extremely low carbon content (~5%) were prepared through the thermobaric treatment of 1,7-bis(hydroxymethyl)-*meta*-carborane under high pressure of 7 GPa and temperature of 1370 K. The microcrystals exhibit a five-fold symmetry and grow in the shape of stars (Figure 7) [142,143]. The unusual shape of the pentagonal microcrystals makes them unique for developing novel micro-machines and semiconductor micro-devices [142].

Heating a mixture of *ortho*-carborane and adamantane (atomic ratio B:C = 5:95) at 8 GPa and 1700 °C results in the formation of boron-doped diamond microcrystals (2–2.5 at.% of boron), whereas only graphite was obtained from a mixture of adamantane and *ortho*-carborane at pressures lower than 7 GPa [144].

## 4. Boron Cluster Anions as Boron Source for Boron-Containing Materials

Coordination chemistry of transition metals with boron cluster anions is one of the most intensively studied fields of boron chemistry [145,146,147]. Research in this area is determined mainly by the fundamental components and concerns metal-boron cluster binding [148], positional isomerism [149,150], and secondary and interligand/inner-ligand interactions in complexes [151,152]. A series of new complex compounds that formed precursors and materials with desired properties were synthesized. Among them are precursors for the low-temperature synthesis of borides and related compounds [153], molecular switches based on a dimeric boron cluster [154], catalysts in the synthesis of organic compounds [155], complexes with luminescent properties [156], copper complexes as models for studying exchange processes and magnetic materials [157], as well as neutron-absorbing materials based on salts of boron cluster anions distributed in the silicate matrix.

Metal borides and related compounds provide ample opportunities for multivariate combination of metal–metal, metal–boron and boron–boron bonds in the resulting phases, thereby providing the possibility of directed changes in their physical, chemical and strength properties [157,158,159,160,161,162]. The most well-known methods of preparing metal borides include: (i) the reaction of metals and boron; (ii) the reduction of metal and boron from oxides when allowing to react with carbon or metals; (iii) the electrolytic reduction of metal and boron from their compounds; and (iv) the thermal dissociation of unstable compounds containing boron and metals. Actually, the processes used to prepare metal borides are often energy-consuming and time-consuming.

In the course of research carried out in our team, we have developed a method for obtaining binary borides during thermal reduction of transition metal compounds [ML*_x_*][An] (M = Co, Ni, An = [B_10_H_10_]^2–^, [B_12_H_12_]^2–^ or [B_20_H_18_]^2–^) with ligands L that can be easily removed at elevated temperature (for example, L = H_2_O, NH_3_, DMF). In the compounds, organic ligands L are considered components, which play the role of organic fuel. Dimethylformamide is one of the most promising substances that can be used as a fuel [163]; its specific heat of combustion (29.652 MJ/kg) is much higher than, for example, that of urea (9.134 MJ/kg), which is often used in SCS processes. The energy capacity of the boron cluster anions themselves makes it possible to lower the boride synthesis temperature, which facilitates the process and reduces energy consumption.

**Figure 8:**
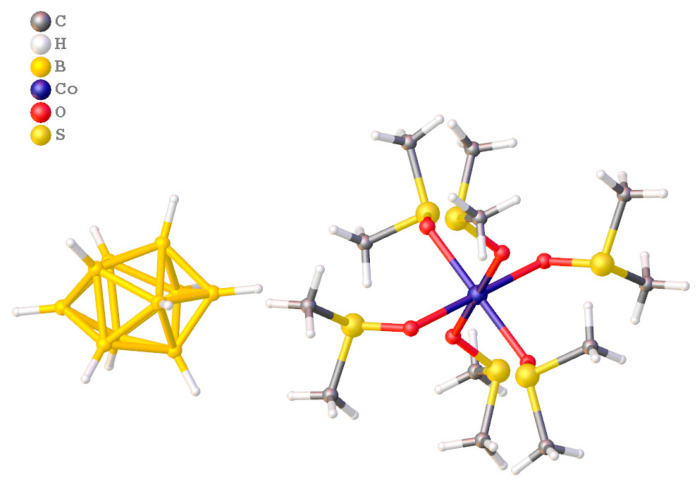
Structures of [Co(DMSO)_6_][B_10_H_10_].

First, we synthesized complexes [Co(DMF)_6_][B_10_H_10_] and [Co(DMSO)_6_][B_10_H_10_] (Figure 8), studied their thermooxidative properties in the temperature range 20–600 °C under argon [164], and determined the annealing temperature. When comparing the IR data of products of thermolysis performed at 600 °C, it was concluded that boride phases were prepared only for complex [Co(DMF)_6_][B_10_H_10_]. The final products were X-ray amorphous that did not allow us to determine the exactly composition of the final products.

**Figure 9:**
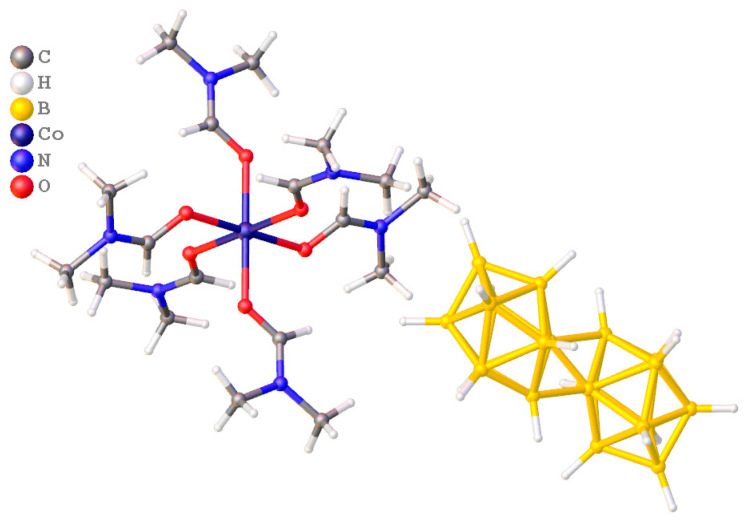
Structure of [Co(DMF)_6_][B_20_H_18_].

When annealing structurally related compounds [Co(DMF)_6_][B_12_H_12_], [Co(DMF)_6_][B_20_H_18_] (Figure 9) and [Co(DMF)_6_][B_10_Cl_10_] in argon at 900 °C [165,166], we succeeded in detecting the CoB phase using X-ray powder diffraction [165]. It was found that for [Co(DMF)_6_][B_12_H_12_], the phases of BN and CoB where prepared in the 1:1 ratio; for [Co(DMF)_6_][B_20_H_18_], a higher CoB:BN ratio but low crystallinity were found; and for the cobalt(II) complex with the decachloro-*closo*-decaborate anion, only CoB was detected. The annealed samples were studied using IR spectroscopy and X-ray fluorescence (for the chloro-containing sample). The nanoparticular character of the decomposition products was shown using TEM.

Thermal reduction of complexes [CoL*_n_*][B_10_H_10_] (L = H_2_O, *n* = 6; N_2_H_4_, *n* = 3) with hydrazine and water molecules in argon at 650 and 900 °C [167,168] resulted in preparation of the dicobalt boride Co_2_B phase as well as orthorhombic and cubic modifications of boron nitride BN. For the aquacomplex, oxide-boride phases were detected. The annealed samples were studied using IR spectroscopy and X-ray powder diffraction. In addition, the samples show different magnetochemical behavior: the oxide–boride phase demonstrated a significant ferromagnetic contribution to the total magnetization of the sample, while the nitride–boride phase had a diamagnetic contribution.

As for structurally related nickel complexes [NiL*_n_*][B_10_H_10_] (L = DMF, H_2_O, *n* = 6; L = N_2_H_4_, *n* = 3) [169], their thermal reduction was studied in the temperature range 20–800 °C in air and in argon. The phases of Ni_3_C and Ni_1 –*x*_C*_x_* were detected using X-ray powder diffraction for annealed complex [Ni(DMF)_6_][B_10_H_10_]; the obtained data indicates that boride-carbide phases were not detected.

Gadolinium tetraboride GdB_4_ was found to form as an only-boride phase by heating a mixture of gadolinium hydride GdH_~2_ and gadolinium *closo*-decaborate Gd_2_[B_10_H_10_]_3_ as a boron source (the boron:metal ratio = 2) at 1400 °C under an argon atmosphere. In a similar way, cerium tetraboride CeB_4_ was prepared from CeH_~2_ and Ce_2_[B_10_H_10_]_3_ at 1100 °C. Using the boron:metal ratio = 6, gadolinium and cerium hexaborides MB_6_ (M = Gd, Ce) were prepared without the coexisting of the corresponding tetraborides at 1200–1400 °C and 1100 °C, respectively. A small number of inclusions (oxides, borates, etc.) can be completely removed using acid treatment with conc. HCI solution [170].

Crystalline ytterbium hexaboride YbB_6_ along with some amount of amorphous boron were prepared by heating ytterbium(II) *closo*-decaborate Yb[B_10_H_10_] in a quartz tube maintained at 10^−5^ Torr to a maximum of 1000 °C [171].

The thermal decomposition of copper(I) *closo*-decaborane Cu_2_[B_10_H_10_] at 800 °C was found to produce crystalline copper boride CuB_24_ and metal copper and amorphous boron [172].

Recently, the annealing of copper(II) complexes with hydrazine [Cu^II^(N_2_H_4_)_3_][B_10_H_10_]·*n*H_2_O or ammonia [Cu^II^(NH_3_)_4_][B_10_H_10_]·*n*H_2_O in argon at 900 °C was used to prepare a Cu@BN boron-containing copper composite [173]. The composition consists of a boron nitride matrix doped with cubic copper(0) nanoparticles with an average particle size of ~81 nm or ~52 nm, respectively.

Modern technology has a high demand for materials which can operate under extremal temperatures. Inorganic polymers attract attention because they offer some properties that are not found in organic materials, such as low-temperature flexibility, electrical conductivity, and nonflammability. The linear polysilicates obtained by the polycondensation of sodium metasilicate with silanol groups are the most widely studied among the non-organic polymers [174].

Prior to studying the distribution of salts of the boron cluster anions in the silicate matrix, the thermal and thermomechanical properties of starting salts (R_3_NH)_2_[B_12_H_12_] (R = Et, Bu) were examined as compared with (Et_3_NH)_2_[B_10_H_10_] [175]. The TGA and DSC data for (R_3_NH)_2_[B_12_H_12_] are similar; thermal destruction is observed at 260–450 °C, and the weakening of intermolecular contacts (softening) is observed before thermooxidative destruction. As for (Et_3_NH)_2_[B_10_H_10_], thermooxidative and thermal destructions occur simultaneously within a narrow temperature range of 260–320 °C, and the softening temperature lies within the range of intensive weight loss.

Furthermore, we studied the thermal behavior of triethylammonium *closo*-decaborate in a silicate matrix [176]. The interaction of sodium silicates of liquid glass (LG) with triethylammonium salts of boron cluster anions was studied in a wide range of component ratios. The compositions formed by addition of different amounts of (Et_3_NH)_2_[B_10_H_10_] (5, 15, 30, 40, 50, 60, and 74 wt%) into sodium liquid glass [176] were studied.

The dissolution of triethylammonium salts of boron cluster anions in sodium LG at room temperature is accompanied by the release of triethylamine, which completely stops when the temperature rises to 100 °C. The absence of a band of stretching vibrations of the NH groups of the triethylammonium cation in the region of 3100–3200 cm^−1^ indicates its complete replacement in the composition by Na^+^ ions. The retention of the formed sodium salts in the silicate matrix is carried out due to the formation of specific cation–anion contacts.

**Figure 10:**
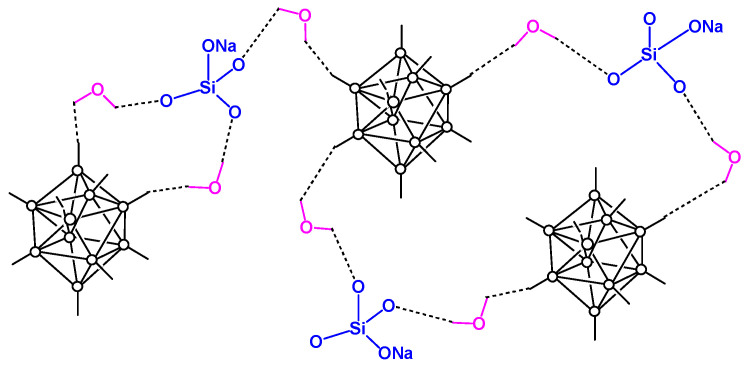
Multicenter interactions between (Et_3_NH)_2_[B_10_H_10_] and components of sodium liquid glass.

It was found that for compositions with *closo*-decaborate anion, the anion oxidation in air begins at 350 °C and is accompanied by a significant exothermic effect. IR spectroscopic analysis of the thermolysis products obtained in air at 350 and 600 °C showed the presence of the *closo*-decaborate anion in the samples [176,177]. A branched 3D system of multicenter bonds between BH-groups of the boron cluster and silanol groups via the water molecules can be assumed in the resulting inorganic polymer composition (Figure 10). The participation of the boron cluster in hydride–proton (dihydrogen) bonds is detected using IR spectroscopy because of the splitting of the band of stretching vibrations of the BH groups ν(BH) observed near 2500 cm^−1^, whereas the formation of hydrogen bonds between sylanol groups and water molecules can be assumed because of the presence of broadened band ν(OH) in the region 3600–3000 cm^−1^. This structure prevents the *closo*-decaborate anion from undergoing complete degradation, thus forming a surface protective layer which consists of borates and silicates, allowing preventing the bulky sample from oxygen diffusion and its further oxidation at high temperatures. The authors concluded that samples are stable up to 600 °C, which is attractive for fabricating boron-rich thermally stable coatings.

In the IR spectra of the compositions, the multiplet splitting of the band of stretching vibrations of BH bonds, which is characteristic of interactions of this kind, is clearly manifested. The thermal stability of individual salts of boron cluster anions is determined by the nature of the anion and cation of the starting compound. The thermal stability of the compositions also depends on the nature of the boron cluster anion. According to the TG and DSC data, the protective layer is formed when the temperature rises to 500 °C during the thermogravimetric analysis. It is worth noting that is that the heat treatment of the sample under these conditions is accompanied by a high exothermic effect, which can lead to the melting of the borosilicate components.

The possibility of using compositions based on the *closo*-decaborate anion as highly heat-resistant boron-enriched materials is evidenced by TMA data [178,179]. The samples are characterized by high heat resistance compared to the original components; they do not soften at temperatures ≥ 600 °C as high thermal and thermomechanical stability is probably ensured due to the formation of a “protective structure” on the surface of the samples during testing, which prevents the diffusion of atmospheric oxygen.

Structural features of boron cluster anions introduced into the compositions have a significant effect on the processes and thermomechanical properties of the compositions.

The formation of a protective layer in the compositions is also observed for the *closo*-dodecaborate anion [180,181]. It was found that heating the composition, in which the amount of the doped component is 60 wt%, leads to the formation of the composite and crystallization of sodium salt of the *closo*-dodecaborate anion on its surface at 200 °C, according to X-ray powder diffraction data [182]. The results of the study of the morphology of the obtained sample by scanning electron microscopy were compared with the results of the morphology of the sodium salt obtained from an aqueous solution. On the surface of the composite, there are needle-shaped nanosized particles with well-formed faces and sizes of 60–100 nm in width and up to 3 μm in length. In a sample of sodium salt obtained from an aqueous solution, only large blocks with a size of about 10–30 μm are present. In addition, when the initial mixture contained 60% of triethylammonium *closo*-dodecaborate at 450 °C, a high plasticization of the composition was noted, as evidenced by the TMA data [181]. The obtained properties may be important for the processing of composites and are probably due to the presence of about 6.6% triethylammonium substituted derivative of the *closo*-dodecaborate anion in the reaction, which is formed during the heat treatment of the composition.

The reaction of LG with the triethylammonium salt of the perchlorinated substituted derivative of the *closo*-decaborate anion proceeds similarly [179,180]. For LG/[B_10_Cl_10_]^2–^ compositions containing the perchlorinated *closo*-decaborate anion up to 20 wt%, their plasticizing properties were determined. In the presence of small amounts of additives, associations formed between the silicate and polyhedral boron anions, which act as crosslinking agents. According to the TMA data, triethylammonium salt of the perchlorinated *closo*-decaborate anion does not soften up to a degradation temperature of 420 °C, whereas in salt (Et_3_NH)_2_[B_10_H_10_], this process occurs at 245 °C. Differences in the deformation stability are also retained in compositions containing equimolar amounts of boron cluster anions. Analyzing the obtained TMA results [179,180], it is obvious that the deformation stability of the system containing the perchlorinated anion is significantly higher compared to that of the decahydro-*closo*-decaborate anion. This fact indicates a more rigid structuring observed in the presence of the perchlorinated anion.

Compositions with a low content of boron cluster anions are of particular interest for studying the structural features of the associates formed. We suggested that the associates formed in the silicate matrix can be distributed as individual particles. which was determined using transmission electron microscopy (TEM). For samples containing the [B_10_H_10_]^2–^ anion, the TEM image shows isolated elongated particles 12.5–47.5 nm in size. In turn, the shape of the particles for the composition with the perchlorinated anion is not so pronounced [180]. In the latter case, particles 5–40 nm in size form agglomerates distributed in a silicate matrix. Thus, it is obvious that the shape and nature of the distribution of associates formed in the silicate matrix directly depends on the nature of the boron cluster anion. As a result of these studies, we have patented a boron-containing neutron shielding material [183], which was obtained by the reaction between sodium silicate Na_2_O(SiO_2_)*_n_* in an aqueous solution of sodium hydroxide with trimethylammonium decahydro-*closo*-decaborate (Me_3_NH)_2_[B_10_H_10_]; the reaction solution was boiled until the trimethylamine formed as a result of the reaction of the sodium hydroxide solution with (Me_3_NH)_2_[B_10_H_10_] is completely removed, then dried by raising the temperature to 300 °C. Due to the numerous supramolecular contacts that appear in the glass structure, the destruction of the [B_10_H_10_]^2–^ anion is not observed up to 600 °C. In addition, the boron content in the product is from 15 to 40 wt%, which provides a high ability of the material to capture thermal neutrons. The obtained neutron-shielding material can be used, in particular, in the encapsulation of radioactive waste, in the creation of protective shields.

Boron-containing compounds can be used as light components for the creation of metal matrix composites [184,185]. The metal composites containing copper and aluminum as matrices and salts Cs_2_[B_10_H_10_], [Me_2_NH_2_]_2_[B_10_H_10_], [Ph_4_P]_2_[B_10_H_10_], [Et_3_NH]_2_[B_10_Cl_10_], Cs_2_[B_12_H_12_], [Et_3_NH]_2_[B_12_H_12_] [149], and [Bu_4_N]_2_[B_12_H_12_] [150] were prepared and coated onto a steel surface. It was shown that the developed metal matrix composites with the boron cluster anion salts can be applied for coatings. A friction cladding method allows one to prepare high-quality coatings, providing a high adhesion of the coating to the metal substrate. No defects were found either in the mass of the coating or on the surface.

## 5. Conclusions

Here, we tried to summarize briefly the synthetic routes and wide application fields of boron-containing materials prepared from boron cluster anions and carboranes. The recent renaissance in chemistry of borohydrides and carboranes is associated with ever-new prospects for their practical use. We hope that the information collected in this article will significantly expand the understanding of the variability of the practical application of boron cluster anions and carboranes to obtain composites and materials based on them, and will provide a novel perspective on the ways to obtain composites with desired properties.

## Data Availability

No new data were created.
